# 34 Artificial Intelligence-Enhanced Multispectral Imaging for Burn Wound Assessment: Insights from a Multi-Centre UK Trial

**DOI:** 10.1093/jbcr/iraf019.034

**Published:** 2025-04-01

**Authors:** Poh Hong Tan, Karl Walsh, Miriam Nyeko-Lacek, Zeeshan Sheikh, Christopher Lewis

**Affiliations:** Newcastle; Manchester University NHS Foundation Trust; Manchester University NHS Foundation Trust; Manchester University NHS Foundation Trust; Northern Regional Burn Centre

## Abstract

**Introduction:**

Accurate burn wound assessment is essential for effective treatment, yet it remains heavily dependent on clinical judgment, which is subjective and prone to error. While various optical-based instruments have been developed to address this issue, their clinical utility has been limited due to the complexity of data interpretation, penetration depth, and feasibility.

The integration of artificial intelligence (AI) with multispectral imaging (MSI) represents a significant advancement. MSI’s ability to collect complex data, providing a deeper and more accurate understanding of wound conditions, combined with AI’s capacity to interpret this data and produce clear, objective, consistent, and easily reproducible outputs.

This study examines the application of AI-enhanced MSI for burn wound assessment in a Multi-centre UK setting.

**Methods:**

We conducted a Multicentre prospective cohort study at the Newcastle and Manchester burn centre, including patients over 18 years old with superficial to full-thickness burns that did not undergo surgery. Multispectral imaging and clinical assessment were performed on admission, and the patient followed up for 21 days. The primary outcome was the reliability and reproducibility of healing prediction, whilst the secondary outcome was the instrument’s feasibility. The AI’s prediction was compared to the clinical healing assessment by 21 days as the reference standard. Image J was used to analyse the images, and the statistical analyses were performed using R (version 4.4.1)

**Results:**

The study included 35 patients and 73 burn images, generating approximately 13 million data points. The mean age of the patients was 51, with an average Total Body Surface Area (TBSA) of 4.06%. Most burns were scalds (n=29). The AI-enhanced multispectral imaging system demonstrated a sensitivity of 80.7% (95% CI: 51.8%–100%) and a specificity of 95.5% (95% CI: 93.3%–97.8%). The overall accuracy of the system was 95.3% (95% CI: 93.2%–97.6%). The mean time from scan to result was five minutes and twelve seconds. The device was portable and utilized in various clinical settings, including clinics, operating theatres, and emergency departments.

**Conclusions:**

Our study demonstrates that the AI-enhanced multispectral imaging (MSI) system offers high accuracy compared to clinical healing outcomes as the ground truth. Its combined attributes of diagnostic precision, operational efficiency, and portability position this device as a transformative tool for revolutionizing current clinical practices in burn wound assessment.

**Applicability of Research to Practice:**

The ideal imaging device for burn wound management must be portable, intuitive, and precise. We present a solution that embodies these attributes while delivering consistent, reliable, and objective results. This innovative device empowers clinicians, enhancing diagnostic accuracy and improving treatment strategies for burn injuries.

**Funding for the Study:**

N/A

